# Cognitive Functioning in Phenylketonuria: A Lifespan Perspective

**DOI:** 10.3390/nu18010146

**Published:** 2026-01-01

**Authors:** Stephan Huijbregts, Cristina Romani

**Affiliations:** 1 Education and Child Studies, Leiden Institute for Brain and Cognition, Leiden University, 2333 AK Leiden, The Netherlands; 2 School of Psychology, College of Health and Life Sciences, Aston University, Birmingham B4 7ET, UK; c.romani@aston.ac.uk

**Keywords:** phenylketonuria, cognition, executive functioning, early childhood, aging, biomarkers, neurodevelopmental disorders, neurodegenerative diseases

## Abstract

Phenylketonuria (PKU) is a hereditary metabolic disorder characterized by the inability to metabolize phenylalanine, leading to neurotoxic accumulation of phenylalanine and significant cognitive impairment. While extensive research has focused on the cognitive outcomes in middle childhood, adolescence, and early adulthood, there is a notable paucity of studies addressing the cognitive functioning of very young and older PKU patients. This review underscores the necessity for further research in these populations, particularly because of the importance of early cognitive development for later cognitive and behavioral functioning and because of the potential implications of PKU and metabolic control for age-related cognitive decline.

## 1. Introduction

Phenylketonuria (PKU; OMIM #261600) is a genetic disorder resulting from a deficiency in phenylalanine hydroxylase, leading to elevated levels of phenylalanine in the blood. Early diagnosis and dietary management are crucial in preventing intellectual disability and other behavioral deficits. Whereas those risks are significantly reduced after early and continuous dietary treatment, many studies have shown persisting deficits in specific cognitive and behavioral domains [1,2,3,4,5,6,7,8,9]. Associations between the level of impairment and Phenylalanine (Phe) levels have also been demonstrated regularly. Accumulation of Phe in the blood and (subsequently) the brain may lead to cognitive impairment and behavioral problems in different ways, with most evidence present for pathways leading to neurotransmitter deficiencies and myelin damage [10,11,12,13,14]. Not many PKU patients over the age of 50, who have been treated early (from birth onwards) and continuously, can at present be found because the introduction of Newborn Screening (NBS), detecting PKU, only took place from the 1970s in most countries. Therefore, there is a fear, supported by preliminary evidence, that cognitive impairments will be exacerbated in older patients: the cognitive decline associated with general aging might be accelerated in PKU patients [15,16]. This fear is mainly based on the overlap in aspects of pathophysiology involved in PKU and dementia-related conditions such as Alzheimer’s Disease (AD) and Parkinson’s Disease (PD). Looking at the other end of the age spectrum, it is well-known that building blocks of cognitive functions already develop during infancy and early childhood (roughly between ages 0 and 7 years), and that early development largely shapes further cognitive development [17,18,19,20]. Therefore, this is another life stage that needs much more attention in PKU research. Another important reason for zooming in on early cognitive development, especially in PKU, is the ongoing discussion on (strictness of) dietary and subsequent metabolic control in different stages of life [21,22,23]. There are some studies in children with PKU showing a direct impact of changing Phe levels (through dietary interventions) on cognitive and behavioral outcomes [24]. In adults, positive effects of interventions (return to diet, dietary or pharmacological intervention) on cognitive outcome measures have also been shown, but clear mediating effects of lower Phe on outcome measures such as cognition and mood are generally absent [25,26,27,28]. Improvements often seem to depend on factors such as years off-diet, Phe levels before starting an intervention, or childhood Phe levels. Also, in adults with PKU, most studies show stronger relations between childhood or lifetime Phe levels than with concurrent Phe levels and cognitive outcomes [29,30,31,32,33], although there are exceptions, potentially related to the aspects of cognition that were assessed [25,33]. Regardless of steady progress and increasing insights into cognitive development and its association with metabolic control in PKU, the current literature highlights a gap in our understanding of cognitive functioning in very young and aging PKU patients. This review aims to explore the implications of PKU on cognitive functioning across different life stages. Context is provided by what is known about the maturing and aging brain and the (combined) influence of certain neurotransmitters, amino acids, and nutrients on these processes.

## 2. Cognitive Functioning in PKU

Cognitive functioning in PKU patients, from middle childhood onwards (approximately those aged 7 and older), adolescents, and adults has been assessed in many different ways, often using standardized tests and questionnaires, and spanning many different cognitive domains. Studies revealed deficits in various domains, most prominently in executive functioning and sustained attention [2,3,6,7,22,23]. Executive functioning, encompassing working memory, cognitive flexibility, and inhibitory control (which, often in combination, contribute to separately defined higher-order executive skills such as problem solving, reasoning/decision-making, planning, and organization), is particularly vulnerable to disruption in PKU patients [2,3,34,35]. Moreover, problems in this domain were shown to extend to mental health problems and lower Quality of Life (QoL), concurrently and over the longer term [36,37,38].

Romani and colleagues performed two meta-analyses, one aimed at adults with PKU and one mainly aimed at children and adolescents with PKU, which also included comparisons to cognitive profiles established in adults with PKU [6,7]. For the meta-analysis focusing on adults, data were included from 26 PKU groups and matched controls, constituting a total of 757 patients (contributing 220 measures for 19 cognitive functions) [6]. Whereas significant impairments were found for IQ and most pre-established cognitive functions, there was significant variability in the magnitude of the effects (group differences compared to healthy controls), with the largest differences found for reasoning, visual-spatial attention speed, sustained attention, visuo-motor control, and cognitive flexibility. Effect sizes were larger with speed than accuracy measures, and with visuo-spatial than verbal stimuli.

In the second meta-analysis, data from 29 (young) adult PKU groups (904 participants with a mean age of 27 years) and 21 child groups (460 participants with a mean age of 11 years) were included [7]. Overall level of impairment was comparable for children and adults, and no clear differences regarding the most strongly affected cognitive functions/domains were evident, although differences were observed regarding the way in which adults and children with PKU performed the cognitive tasks. In adults, performance speed was more strongly affected, with accuracy of performance more often preserved, whereas in children with PKU, this frequently was the other way around. In both age groups, both concurrent and historical blood Phe level modulated the effect size of the impairment. The fact that concurrent Phe influenced cognition in adult PKU patients supports the notion of continuity of the influence of Phe. In most studies, however, historical Phe levels had an even larger influence on later outcomes than concurrent Phe levels, which supports the notion of developmental continuity of Phe-related cognitive impairments that arose earlier in life [29,30,31,32,33].

Studies have shown that executive functioning develops in stages throughout childhood, with inhibitory control the first to emerge and working memory, and particularly cognitive flexibility, having prolonged developmental trajectories [39]. This, in turn, could influence the periods of time where, in PKU, metabolic control has the greatest impact [32]. Considering the evident variation between individuals in cognitive development, it is customary to utilize age ranges to mark boundaries of different developmental stages. A matter that further complicates the demarcation of developmental stages for EF is the so-called ‘unity and diversity’ of the construct [40,41]. As noted before, decision making, problem solving, reasoning, planning, and organization, which do not fully develop before people are in their 20s, are often listed as separate executive functions, but are in fact “built” from the core executive functions, inhibitory control, working memory, and cognitive flexibility. This illustrates the unity (they all stem from the same core executive functions) and subsequent developmental continuity of EF, but also the diversity of EF as different combinations of core executive functions, or of EF with other aspects of cognition, lead to different demands of the brain [40]. The combining of different functions could constitute a separate stage or stages in cognitive development [42]. Furthermore, when attempting to determine developmental stages for EF, the possible involvement of motivational/emotional aspects should be considered. Generally, emotional maturity is reached only in the third decade of life, and it is well-known that such emotional/motivational components influence task performance and involve more brain regions than core executive functions by themselves [40,42,43,44]. Practically, health care professionals generally prefer to work with more clearcut boundaries, which can also be seen in international guidelines for the treatment and monitoring of PKU, where childhood (0–12 years), adolescence (13–18 years), and adulthood (>18 years), or sometimes only childhood and the period beyond 12 years, are distinguished [21,23]. Even using such broad subdivisions of developmental stages, there are many longitudinal studies showing developmental continuity of EF as well as translational effects of childhood EF on mental health, adaptive functioning, and quality of life during adolescence and adulthood [42,45,46,47,48,49]. For PKU specifically, it has also repeatedly been shown that childhood and adolescent phenylalanine levels are very predictive of later cognitive and mental health outcomes, with, on a number of occasions, historical metabolic control being more predictive of adolescent or adult outcomes than concurrent metabolic control [4,29,32,33,50]. It should be noted, though, that several studies have shown nuances to straightforward historical and concurrent metabolic control—cognitive outcome associations. For example, fluctuations in Phe levels, sometimes in specific, later age periods, were shown to have a greater, or at least independent influence on cognitive outcomes beyond absolute or average Phe levels [51,52]. Such differential influence may also depend on which aspects of cognition were examined.

Perhaps the main challenge of the meta-analyses was the large number of different tasks to assess certain cognitive domains and functions, resulting in 278 separate outcome measures available for adults and 175 for children [6,7]. There is generally good evidence for the claims that tasks measure particular cognitive functions, but the sheer number of available tasks complicates matters. First, there is the definition of EF: is the narrow definition used, with the core executive functions inhibitory control, working memory, and cognitive flexibility, or a broader definition which includes higher-order executive functions such as decision-making, reasoning, planning, and organization as well? Is attention included in the definition? There is overlap between attention and executive functions, e.g., divided attention (specifically attention switching) overlaps with cognitive flexibility, and focused attention (ignoring “irrelevant” distraction) has an overlap with inhibitory control. There are, however, differences as well, for example in (the number of) active brain regions during attention and EF tasks and the extent to which certain neurotransmitters are used. For example, evidence indicates a greater requirement of dopamine during EF tasks and of norepinephrine during (sustained) attention tasks. A third consideration is that the design of different neuropsychological tasks could lead to differences in the extent to which the cognitive functions of interest are measured. Additionally, they might tap into multiple cognitive functions simultaneously, which can further influence task performance in itself [53].

In order to find out whether the tasks can be used interchangeably, many cross-validation studies would have to be performed. For this purpose, the same PKU patients and healthy controls perform different tasks claiming to measure the same domains and find out the extent to which performances correlate, as well as the extent to which performances of the PKU patients relate to historic and concurrent metabolic control. One recent study did this with tasks from the Cambridge Neuropsychological Automated Test Battery (CANTAB) and the Amsterdam Neuropsychological Tasks (ANT), both of which have often been used in PKU research. The study focused on (sustained) attention and EF and found good agreement between tasks claiming to measure similar domains/functions [54]. Whereas this is positive news when new studies or clinical trials are initiated, or when longitudinal cognitive monitoring is introduced, it seems infeasible to perform cross-validation studies for all instruments that are out there and have been used to assess, for example, executive functioning. However, one could argue that at least the most common/frequently used instruments should be cross-validated against each other.

A selection of such instruments was described in recent guidelines and other papers [23,55]. Although largely because of a lack of cross-validation studies, these selections of instruments are not necessarily optimal, they provide a rather good representation of the most important cognitive domains in PKU research and monitoring.

Neuropsychological tasks that have been used to assess executive functions (in PKU and in general) include paper-and-pencil tasks and computerized tasks that seldomly purely and only assess inhibitory control, working memory, or cognitive flexibility. As noted, however, in PKU, the tasks that are most sensitive require a combination of executive functions or of executive functions with other aspects of cognition [2,3,7,32,53].

For inhibitory control, go-no-go/stop signal tasks or flanker/distraction tasks generally do not show large Phe-related differences between PKU patients and their healthy counterparts, but tasks combining inhibitory control and sustained attention (e.g., several different dots/continuous performance tasks) do [2,32]. It is also more likely to find effects when ‘pure’ inhibition tasks are paced, in other words, they additionally require motor speed. For working memory, it appears that tasks do not require other cognitive abilities to the same extent as inhibitory control to demonstrate Phe-related impairment in PKU patients, although whether or not a task is paced is also an important component here (e.g., Paced Serial Addition Test (PASAT), Rapid Visual Information Processing tasks (RVP/RIVP) as in the CANTAB, several N-back tasks, Memory Search_ 2 Dimensions from the ANT, List Sorting Working Memory tasks, as in the National Institute of Health (NIH-)Toolbox [55,56], and several Spatial Span/Visuospatial Sequencing Tasks, as in the CANTAB and ANT). Another important factor to distinguish PKU patients and healthy controls or to show associations with metabolic control is the working memory load, whether this relates to the complexity of the stimuli that have to be retained and processed in working memory or the number of items that have to be retained and processed. Rapid Visual Information Processing tasks are examples of working memory tasks where WM-load (length or complexity of digit target sequences that have to be memorized) can be manipulated, but also the length of the task, thereby increasing or decreasing the sustained attention component. Obviously, this can also be done with other tasks, but this underlies the fact that RVP tasks are often considered to be more of a measure of sustained attention than of working memory. Other theoretical considerations need to be taken into account as well when defining tasks as working memory tasks. Working memory contains a consolidation/maintenance component and a manipulation/monitoring/updating component (e.g., grouping, linking with information in long-term memory, etc.). The latter is generally more demanding than just keeping the offered information active (the maintenance component), requiring more communication between brain regions. Tasks such as the California Verbal Learning Task (CVLT) and the Rey Auditory Verbal Learning Test (RAVLT) are generally used to assess long-term memory/learning, but also tap into the WM-maintenance component. These are therefore less likely to distinguish PKU patients and healthy controls, whereas, for example, the Visuospatial Sequencing or List Sorting Tasks ask participants not only to recall certain items but to recall them in a specific order or according to a specific categorization.

Finally, PKU patients have frequently been shown to be vulnerable regarding cognitive flexibility tasks. Here again, however, there are significant differences in complexity between tasks. Tasks such as Digit Symbol Coding, Trail-making tests (B-A) (TMT), Stroop tasks, and verbal and semantic fluency tasks are ‘easier’ than tasks such as Shifting Set—Visual (SSV, from the ANT), the Multitasking Test (MTT, from the CANTAB), and the Dimensional Change Card Sort (DCCS, among others from the NIH-Toolbox), and therefore less likely to show PKU-related differences or impairment. All aforementioned tasks involve switching between response rules, but the SSV, MTT, and DCCS are again paced and/or involve unpredictable switches within task parts. It can also be argued, however, that a task such as the TMT could be considered to have an additional demand, i.e., visuo-motor coordination, as does, for example, the Pursuit task from the ANT, and motor functioning/control is also related to the dopamine depletion hypothesis for PKU [53,57].

Earlier, we mentioned that cross-validation of different tests or test batteries measuring or claiming to measure the same constructs would be very helpful for future studies, particularly longitudinal and/or clinical studies. We also acknowledged that it would be very ambitious to include all available tasks, even if only the ones measuring the most vulnerable domains in PKU were selected. One test battery that should definitely be linked to the ANT and CANTAB is the NIH Toolbox [55,56]. In the ANT, CANTAB, and NIH-Toolbox, tasks were assembled and computerized that were often available as paper-and-pencil tasks before, or variants have been designed. The paper-and-pencil tasks have not disappeared and some researchers recommend picking paper-and-pencil tasks to measure the core impaired domains in PKU for assessment and monitoring (over the computerized tasks) [58], but the only advantage there would probably be lower costs: the computerized tasks provide more information, particularly when performance speed and subsequent potential speed-accuracy trade-offs play an important role besides performance accuracy.

## 3. Cognitive Functioning in Young PKU Patients

The majority of studies into childhood cognitive functioning in PKU are aimed at children aged 7 years and older. This can be explained by methodological constraints, more specifically by the fact that neuropsychological testing is much easier and offers a much more refined picture from approximately that age onwards. Studies that did include data from children below the age of 7 mostly focused on Phe levels during infancy and early childhood, predicting later cognitive outcomes, or, when they did perform neuropsychological assessments early in life, on general intelligence/IQ [59,60,61]. Specific cognitive domains in young PKU patients have been addressed much less frequently. An exception is a study by Paermentier and colleagues, who examined 23 children with Hyperphenylalaninemia (HPA), 12 with classical PKU, and 11 with Moderate Hyperphenylalaninemia (MHP), aged 3 to 5 years, and compared their performance on a series of EF tasks to that of 50 control children [62]. They found that preschool MHP patients (who have lower Phe levels than PKU patients) had comparable executive scores to control subjects, whereas PKU patients scored significantly worse on 3 EF tasks, measuring verbal working memory, visual working memory, and cognitive inhibition. Furthermore, several correlations were identified between EF scores and Phe levels at inclusion, mean Phe level, and variability of Phe levels throughout life. Whereas these findings provide support for the notion that Phe-related EF impairment can already be found in very young PKU patients, results also showed that it is very important to clearly establish which aspects of executive functioning are being assessed and which instruments are used for those purposes. For example, when assessing executive functioning in daily life, using the Behavior Rating Inventory for Executive Function (BRIEF)-Preschool questionnaire (filled out by parents and teachers), no differences with healthy controls were found [62]. The BRIEF is an increasingly popular instrument to assess executive functioning in daily life, and even though it has been validated against other questionnaires measuring mental health and daily life functioning as well as some selected laboratory measures (i.e., neuropsychological tasks), there is evidence for a lack of overlap with tasks addressing the core executive functions as discussed in this paper [63,64]. This does not mean that BRIEF is an insufficient instrument, just that it measures something different compared to the neuropsychological EF tasks.

Measuring executive functioning in young children, particularly from infancy to six years of age, involves a range of standardized assessments and observational tools. Some of the aforementioned tasks used for older children, adolescents, and adults to assess inhibitory control, working memory, and cognitive flexibility have also been administered to younger children, e.g., the Dimensional Change Card Sort (DCSS-) task to measure cognitive flexibility [65,66]. Generally, however, tasks need to be adapted for children aged 6 and younger (see, for example, [54] for ANT- and CANTAB tasks that could not really be successfully finished even by 7-year-olds). Still, quite a few tasks have been developed that measure the core executive functions. Examples of working memory tasks for young children that require them to remember and utilize or update information are variants of “Hide-and-Seek” tasks [67] and (shortened or with fewer items) span tasks, while examples of inhibitory control tasks are the “Marshmallow Test”, which specifically measures the ability to delay gratification, the Sun-Moon Stroop Task, and the (Luria’s) Hand Game [68]. In their study, Paermentier and colleagues used the Sun-Moon Stroop Task (based on the Day-Night, see also [69]), where, in the control phase, the child is asked to name the pictures as shown and, in the inhibition phase, the child must prevent the predominant response and say “sun” when they see a “moon” or vice versa as quickly as possible), and the Hand Game (in the control condition the child is asked to reproduce the examiner’s gesture, a fist or pointed finger; in the inhibition phase the child performs the opposite gesture) to assess verbal and motor inhibitory control. In order to assess working memory, they used the Digit Span (forward and backward) and the spatial span test, where, on a board with 10 randomly placed cubes, the child points to a series of cubes in the order indicated by the examiner (forward spatial span) and then in the opposite order to that presented by the examiner (backward spatial span). In terms of measurement potential, some tasks for very young children have been defined as both inhibitory control and working memory tasks. A previously mentioned example is the Day-Night or Sun-Moon Stroop task, where two, albeit connected, rules have to be memorized, which fits with working memory, but the ‘natural’ or automatic response has to be suppressed, which fits better with inhibitory control. Another example is the “Simon Says…” task in its different variants, where only when Simon says, for example, “touch your nose”, it has to be touched, and when someone else says the same thing, the response has to be suppressed. So again, two rules have to be memorized, which is closer to WM, while a relatively automatic response has to be suppressed in a number of cases, which is closer to inhibitory control [70]. The DCCS, which also features in the NIH Toolbox [57,68], and which requires shifts in responding according to one of two dimensions (shape or color) was used to assess cognitive flexibility by Paermentier and colleagues, as well as the Brixton test for preschoolers [71], which, in this case, involved thirty clay pots placed in a line, with one (to be determined by the child) hiding a mouse, with three switches (+1, −2, +3) in the hiding location.

Finally, Paermentier and colleagues measured affective decision making, which was assessed with the Children’s Gambling Task (CGT), where children, with each trial, had to pick one of two decks/sets of cards [43]. One could be advantageous incidentally, but the other (leading to more gains eventually) was more advantageous in the long term. Importantly, the development (independent of IQ) of the cognitive abilities required for this task was shown to be between ages 3 and 4, with 3-year-olds also performing above chance level. Therefore, partial evidence supporting the claim that the NIH-Toolbox is suitable for 3-to-85-year-olds [66] has been provided. Three years does appear to be the youngest age at which somewhat accomplished executive functioning can be measured, although tests measuring very rudimentary aspects of EF have been developed for even younger children as well.

For example, to test very early inhibitory control (as early as 12 months of age), several variants of the “don’t paradigm” have been developed [72,73]. Basically, these tasks assess how well the infants/toddlers comply with (mothers’) instructions (such as “no-no” and “don’t do that”). In this phase of life, executive functioning is often called self-regulatory behavior, which has been shown to be uniquely predictive of behavior problems and academic achievement later in life [45]. Neuropsychological assessment, especially of EF, has, to date, been missing from most studies investigating very young PKU patients (for exceptions, see Diamond et al. [74] and Arnold et al. [75], whose youngest participants were 6 and 12 months old, respectively). In order to gain insights into cognitive development at such ages, it is likely that the focus should lie on verbal abilities and motor skills, both of which have been shown to predict later cognitive (EF-) development.

## 4. The Roles of Early Verbal Abilities and Motor Skills in Cognitive Development

Whereas EF appears to be the most strongly affected cognitive domain in PKU, it is important to note that other cognitive abilities should be assessed as well, particularly during infancy and early childhood. Early language/verbal abilities and motor skills are very predictive of cognition, including executive functions, later in life. For early language/verbal abilities, for example, large longitudinal studies showed that such abilities, including vocabulary size, syntactic complexity, and phonological awareness, not only predicted linguistic outcomes later in life, but also executive functioning (working memory), social cognition/communication, academic achievement (including mathematical performance), and further societal outcomes [18,19].

With respect to motor skills, significant associations exist between motor development in infancy and early childhood and subsequent cognitive abilities. Several longitudinal studies provide evidence supporting this relationship. For example, in the Helsinki Birth Cohort Study, it was found that individuals who achieved walking at an earlier age showed greater cognitive ability in early old age, an association that persisted after adjusting for socioeconomic and demographic factors [20]. In the Groningen Prospective Cohort Study, infants with lower motor variation and performance scores, assessed using the Infant Motor Profile (IMP) at 4, 10, and 18 months, had IQ scores that were, on average, almost 9 points lower at age 4 compared to those with typical motor scores [76]. In the Mexican Preschoolers study, positive associations were observed between early motor skills—such as stationary balance, grasping, and visual-motor integration—and later cognitive abilities, including verbal, quantitative, and memory skills [77]. And, in a final example, Bornstein and colleagues [17] performed a 14-year longitudinal study, showing that infants exhibiting advanced motor-exploratory competence had higher intellectual functioning in childhood, which subsequently contributed to better academic achievement in adolescence.

There are some studies in PKU that have assessed neuromotor development in very young patients, i.e., in infants/toddlers with PKU. The Wechsler Preschool and Primary Scale of Intelligence (WPPSI)—4th Edition [78] can be used from 2.5 years old whereas, for example, the Bayley Scales of Infant and Toddler Development, can already be used from 1 month of age [79]. The Bayley Scales are used to assess five domains: cognitive (attention, problem-solving, memory, object permanence, and concept formation), language (receptive and expressive), motor (fine and gross, e.g., grasping, crawling, walking), social-emotional (engagement, emotional expression, interaction with caregiver), and adaptive behavior (daily life functioning, usually through caregiver questionnaire) [79]. When this was assessed in PKU, results generally showed some direct or indirect evidence for associations with metabolic control [80,81], although studies extending over longer periods of time (which could show longitudinal links with, for example, EF-development) are currently still missing.

The body of scientific evidence clearly supports the notion that early language and motor abilities serve as robust predictors of later cognitive development. Therefore, a focus on those abilities in PKU research when children are very young would be recommended.

## 5. Aging and Cognitive Decline in PKU

As noted before, not many studies including neuropsychological assessments in PKU patients over the age of 50 have been performed, as patients treated from birth onwards following the introduction of NBS for PKU have generally not reached that age yet. Still, as individuals with PKU age, the risk of cognitive decline may be heightened [82].

One study compared cognition and well-being between middle-aged adults with PKU (average age 45.8 years) and younger adults with PKU (average age 26.7 years) [16]. The findings indicated that the middle-aged group exhibited higher levels of anxiety and depression compared to age-matched controls, as well as larger cognitive impairments and a steep deterioration of quality of life compared to younger adults with PKU (steeper than expected from controls). On the other hand, compared to age-matched healthy controls, the size of impairment (in, among others, EF and sustained attention) was not larger (in some instances even smaller) for the middle-aged group. The authors recognized there were quite a few limitations to their study, among others the different testing circumstances for the young adult and middle aged groups (only the middle-aged group was tested during the COVID-pandemic) and the small number of participants, to which one could add the high number of neuropsychological tests and associated cognitive domains and the lack of metabolic data: possibly participation of only relatively well-controlled middle-age PKU patients led to a selection bias. As noted, the authors recognized these limitations and called for more research into this topic [16]. Feldmann and colleagues, who conduct a longitudinal follow-up study of a cohort of German PKU patients, reported stable cognitive impairments among middle-aged patients (median age during latest test round: 47 years), with persistently poorer performances among the older compared to the younger patients within their cohort. The authors hypothesize this might be related to an earlier relaxation of the Phe-restricted diet among those patients [30,31]. Further indirect evidence for accelerated aging in PKU stems from a study by Pilotto and colleagues [15]. In this study, 19 PKU patients (median age 41 years) and healthy, matched controls were compared on a wide range of measures, including those measuring brain integrity and cognition. For 10 patients, cerebrospinal (CSF-) concentrations of neurodegenerative biomarkers were included. In addition to cognitive problems and atrophy in the putamen and right thalamus, the authors reported higher β-amyloid 1–42, total tau, and phosphorylated tau levels in PKU patients compared to controls. Moreover, they reported significant correlations between plasma Phe levels and the number of failed neuropsychological tests and parietal brain atrophy. No significant correlations were reported between plasma Phe, biomarkers for neurodegeneration, and cognition, possibly due to low numbers of participants with complete data (who were obviously also still quite young for the detection of age-related cognitive decline). Such correlations would have substantiated the link between PKU and accelerated aging further. Nonetheless, these results already provide a good basis for further research into this potential association.

Another reason, obviously, to pursue such research further is the relatively widespread worries among patients themselves [82,83]. Several issues need to be taken into account. First of all, the ages (and circumstances) at which PKU patients should undergo (additional) neuropsychological testing. The latest European guidelines recommend that neuropsychological assessment is offered at the start of formal education (4–6 years), at 12 years of age as, in Europe, this is the age when the upper target blood Phe level changes to 600 µmol/L and adherence to dietary treatment may decline, at 18–20 years when there will be increasing independence and a transfer to adult clinical services, and every 5–10 years thereafter [23]. Knowledge of the importance of early cognitive development suggests that neuropsychological testing should start even earlier, while much is still to be done to achieve recommendations for later assessments as well. In practice, neuropsychological assessment in PKU still mainly takes place for research purposes and is not yet fitted to detecting potentially accelerated cognitive aging. Moreover, neuropsychological testing during (older) adulthood is generally more focused on attention and memory than on the core EF-deficits established in PKU, and mainly takes place when individuals are first presenting with symptoms of dementia or start worrying themselves about possible deterioration.

Early- or young-onset dementia (EOD/YOD) is typically defined as dementia diagnosed before the age of 65, and many risk factors have been associated with it. For instance, in a large UK Biobank study, increased risk of YOD was associated with low socioeconomic status, apolipoprotein E status, alcohol use disorder, social isolation, vitamin D deficiency, high C-reactive protein levels, hearing impairment, orthostatic hypotension, stroke, diabetes, heart disease, and depression [84]. Obviously, the risk factors are those that are most prevalent in the general population, so relatively rare metabolic disorders such as PKU are highly unlikely to appear as risk factor for EOD/YOD themselves.

Still, some of these risk factors (which individually have been related to cognitive impairment as well), such as social isolation and depression, vitamin deficiencies, and diabetes, may be more prevalent among PKU patients [85]. This, in turn, may exacerbate the risk of EOD/YOD in this population further, on top of the PKU-related pathophysiology, i.e., the impact of prolonged exposure to high Phe levels on brain integrity, neurotransmitter levels and cognition/mental health.

Moreover, a potential risk factor that seems to be missing is cognitive functioning as a child/adolescent, while longitudinal studies have demonstrated considerable cognitive stability throughout life [86]. Research has specifically explored the relationship between early-life cognitive performance and the risk of developing dementia in later years. Results from the Project Talent Aging Study, which followed individuals from high school for nearly 60 years, for example, indicated that general cognitive ability during adolescence predicts the risk of cognitive impairment and dementia in later life [87].

Cognitive assessment is crucial for detecting early signs of dementia. As noted, attention and memory are the cognitive domains featuring most prominently in assessments of dementia. Commonly used assessments include the Mini-Mental State Examination (MMSE) [88], a brief screening tool that assesses cognitive function through questions related to orientation, attention, memory, and language, the Montreal Cognitive Assessment (MoCA) [89,90], which evaluates different cognitive domains and is sensitive to detecting mild cognitive impairment (MCI), and the Addenbrooke’s Cognitive Examination III (ACE-III) [89,91]. The instruments are quite similar, have generally been cross-validated, and are often supplemented by informant-rated questionnaires for diagnostic purposes [92]. Certain instruments (MoCA and ACE-III) appear more sensitive to picking up MCI or EOD/YOD, which may be important when (standardized) instruments are chosen in PKU research. All instruments cover, to some extent, the core EF-functions that were shown to be particularly vulnerable in PKU. For example, the MoCA includes the Trail Making Test, Digit Span forward and backward, Delay Recall, a serial subtraction task and a verbal fluency task, which may all be considered measures of core executive functions. It also includes a vigilance/sustained attention task, which, as noted, is also an aspect of cognition shown to be sensitive in PKU patients of all ages. Some of these tasks also feature in ACE-III, whereas MMSE is shorter and less focused on EF. For monitoring and potential diagnoses of EOD/YOD in older PKU patients, it is probably important to add some pure memory tasks (assessing short- and long-term episodic memory using recall and recognition paradigms) and some pure visuospatial and motor tasks (e.g., orientation and drawing tasks) to the core EF- and sustained attention tasks. Moreover, for some core executive functions, such as inhibitory control, some short tasks may have to be added as this has been shown to be a highly vulnerable EF (perhaps even more so when containing a motivational/emotional or motor component) in both dementia and PKU [41,53,93,94].

Please see Table 1 for a non-exhaustive overview of which neuropsychological tests can be used to assess executive functioning across the lifespan in PKU.

## 6. Biomarkers for Cognition: Commonalities Between PKU and Neurodevelopmental Disorders

As noted, PKU has been associated with relatively low levels large neutral amino acids (LNAAs) like tyrosine (Tyr) and tryptophan (Trp) and there is evidence showing that elevated blood phenylalanine (Phe) competitively inhibits transport of Tyr and Trp across the blood–brain barrier, which could lead to reduced cerebral levels of dopamine and serotonin (as Tyr and Trp are their respective metabolic precursors) [9,13,22,95]. Please see ref. [22], page 2, for clear schematic depictions of the phenylalanine hydroxylating system and the pathophysiology of PKU.

Reduced or imbalanced dopamine and serotonin levels, albeit caused by different neurobiological processes, have been linked to cognitive impairments, resembling those most frequently observed in PKU, in a wide range of neurodevelopmental disorders and neurodegenerative diseases. For example, in attention-deficit/hyperactivity disorder (ADHD), the defining executive function and attention deficits have been related to alterations in dopamine transporter function and dopamine signaling, as well as genetically-determined serotonergic abnormalities [96,97,98]. Studies have pointed out a dynamic interplay between monoaminergic systems, sex differences, and only specific DA-based therapeutics, revealing DA-influences on the ADHD-phenotype as potential explanations for inconsistent results. Also, less well-known neurodevelopmental disorders with a clearer neurobiological, genetically determined basis than ADHD often involve dysregulation of dopaminergic and serotonergic systems, which, subsequently, have been related to cognitive and behavioral phenotypes characterized by executive dysfunction. These include rare genetic disorders such as tyrosinemia type 1 (TT1, where the deficit is expressed in a lack of tyrosine breakdown), aromatic amino acid decarboxylase deficiency (AADC, abnormalities in dopamine and serotonin synthesis), tyrosine hydroxylase deficiency (affecting dopamine synthesis), and BH_4_-metabolism disorders. These include GTP cyclohydrolase I (GTPCH), 6-pyruvoyl-tetrahydropterin synthase (PTPS), Sepiapterin reductase (SPR), and DNAJC12 deficiency, affecting tetrahydrobiopterin synthesis with broad monoamine deficits: DA, 5-HT, NE) [99]. In contrast with PKU, most of these disorders do not feature elevated phenylalanine levels, but they all have disrupted tyrosine metabolism. Shortages of tyrosine may lead to shortages of catecholaminergic neurotransmitters, but tyrosine levels that are too high are likely to be toxic as well, i.e., production of catecholaminergic neurotransmitters may also be too high and turn out to be counterproductive with respect to cognitive (executive) functioning [100,101,102].

Thus, both reduced and excessive tyrosine and catecholamines could affect cognitive functioning in a negative way, but, in PKU, there are also shortages in the brain of other LNAAs (e.g., tryptophan for serotonin production) and there is excessive Phe, which, in turn, might affect brain structure and damage myelin [11,12,13,14,103,104]. In PKU, structural and myelination changes correlate with blood and brain phenylalanine levels and cognitive deficits, particularly with impaired executive functioning. Thus, structural and myelin changes may be considered another biomarker for impaired cognition in PKU. Again, these are not unique biomarkers to PKU. An example of a neurodevelopmental disorder where imaging studies have shown volumetric gray matter and white matter (i.e., myelin) abnormalities in brain regions (or parts of the brain connecting those regions) associated with executive functioning is neurofibromatosis type I (NF1), a genetic disorder characterized by RAS-protein overactivity [105,106]. The convergence in MRI-, especially diffusion tensor imaging (DTI)-visible abnormalities, such as reduced fractional anisotropy and altered mean diffusivity in fronto-striatal brain networks, across PKU and, for example, NF1, suggests shared vulnerabilities in the neural circuits underpinning executive functioning and motor control.

It may be concluded that all these conditions—ranging from rare metabolic enzyme defects and other genetic disorders to idiopathic neurodevelopmental syndromes—share functional neurotransmitter abnormalities and altered brain structures that correlate with EF and (sustained) attention deficits, allowing biomarker-guided diagnosis and potentially targeted intervention.

## 7. Biomarkers for Cognition: Commonalities Between PKU and Neurodegenerative Diseases

The dopamine and serotonin hypotheses provide insights into the neurobiological underpinnings of cognitive impairments in PKU. Elevated phenylalanine levels can disrupt the synthesis and metabolism of these crucial neurotransmitters. Research suggests that reduced serotonin and dopamine levels may contribute to mood disorders and cognitive dysfunction in PKU patients, although direct links have only sparsely been shown [10,14,15]. For example, Pilotto and colleagues reported lower plasma and CSF 5-hydroxyindoleacetic acid (5-HIAA, serotonin metabolite) and 5-hydroxytryptophan (5-HTP, serotonin precursor) in ten early-treated PKU patients (mean age 38.2 years) compared to 15 age-matched controls [14]. Together with homovanillic acid (HVA, dopamine metabolite) levels, these were negatively related to Phe levels. Moreover, a decrease in 5-HIAA, 5-HTP, and HVA levels correlated with voxel-based morphometry-determined atrophy (in precuneus, frontal, and occipital areas, respectively). A link with cognition or mood was, however, missing. In a series of articles from another group, relations between Phe, brain atrophy (WM), and cognition were shown, but there, the biogenic amines (monoamines) are missing from the equation [12,107], as they are from Pilotto and colleagues in the 2021-article [15]. Boot et al. [10] and Manti et al. [108] show links between the amines and cognitive outcome measures. In Manti et al., associations were observed between peripheral biogenic amine measures, including 5-HIAA, 5-HTP, and 3-O-methyldopa (3-OMD, dopamine precursor metabolite), which were lower in PKU patients than in controls and related to concurrent Phe levels, and BRIEF-scales. Such associations were also observed for a Phe-related index of dietary control (IDC) and tyrosine, but not for concurrent Phe, leading the authors to conclude that the peripheral biogenic amines could potentially be seen as biomarkers for cognition in PKU that were independent of concurrent Phe. In Manti et al. and Boot et al., the brain atrophy/demyelination link was not investigated with DTI/MRI. However, in Boot et al., another imaging technique, Single Photon Emission Computed Tomography (SPECT), was used to assess striatal dopamine (D2/D3) receptor availability. This was higher in PKU patients (N = 18, mean age 31.2 years) (which was considered indicative of lower cerebral dopamine levels), and receptor availability was inversely related to performance on a cognitive flexibility task and positively related to impulsivity, i.e., inhibitory control in daily life. Moreover, the PKU patients had lower plasma HVA and 3-methoxy-4-hydroxy-phenylglycol (MHPG, norepinephrine/noradrenaline metabolite) levels, the predominant metabolites of dopamine and norepinephrine, respectively, and higher impulsivity compared to healthy controls [10].

Alzheimer’s and Parkinson’s diseases are neurodegenerative diseases with distinct pathological hallmarks but overlapping neurotransmitter dysfunctions. AD is primarily characterized by amyloid-beta (Aβ) plaques, tau neurofibrillary tangles, and cholinergic degeneration [109,110], whereas PD is marked by dopaminergic neuron loss in the substantia nigra and Lewy body pathology [111,112].

Beyond these well-established changes, disruptions in multiple neurotransmitter systems have been implicated in the cognitive and motor symptoms (e.g., bradykinesia, tremors, and rigidity) of both diseases [113,114,115]. For example, both AD and PD show a decline in serotonin (5-HT) and norepinephrine (NE), particularly in the serotonin-producing raphe nuclei and the norepinephrine-producing locus coeruleus [113]. As noted, serotonin is strongly involved in cognition and mood regulation, and norepinephrine is very important for attention and arousal.

Grey and white matter abnormalities have also clearly been established in different neurodegenerative diseases [116,117,118]. Neurodegenerative diseases such as AD and PD are primarily associated with neuronal loss, i.e., they have traditionally been characterized as grey matter disorders. However, myelin and white matter degradation are increasingly recognized as key pathological features. Myelin loss can lead to impaired neuronal communication, exacerbating cognitive and motor dysfunction. In Alzheimer’s Disease, postmortem analyses have revealed significant myelin loss in the hippocampus, corpus callosum, and frontal white matter. Imaging studies using diffusion tensor imaging (DTI) demonstrate reduced fractional anisotropy (FA) and increased mean diffusivity (MD), indicative of WM integrity loss [118]. Oligodendrocytes, the primary myelinating cells in the CNS, show signs of degeneration in AD. Amyloid-beta (Aβ) plaques and tau pathology are implicated in disrupting oligodendrocyte function, leading to impaired myelin repair.

PD is characterized by dopaminergic neuron degeneration in the substantia nigra; however, recent studies suggest concurrent myelin loss in the nigrostriatal tract. DTI studies have identified significant white matter disruptions in PD, particularly in the corpus callosum, internal capsule, and corticospinal tract [119]. The evidence to date indicates the significant role of myelin deficiency and white matter degeneration in both PKU and neurodegenerative diseases, such as AD and PD. Myelin loss in AD contributes to cognitive decline, while in PD, it exacerbates motor dysfunction. As noted, in PKU, some associations with cognitive functioning have also been reported. As with the choice for specific neuropsychological assessment instruments, the exact choice of biomarkers in PKU clinical and experimental follow-up studies should also be examined further. A good example is the DTI-MD measure. Whereas in AD and PD increased MD is considered indicative of damaged WM, in PKU, there are several reports of reduced MD [11,107]. DTI-FA, where a reduction is consistently considered indicative of damaged WM, therefore seems to be a more reliable biomarker option. Whereas the clinical relevance of these potential biomarkers needs much more study, as well as the exact mechanisms leading to white matter damage/demyelination (including genetic predisposition, environmental triggers, immune dysregulation, neuroinflammation, and alterations in the gut-brain axis [120]), the overlap in brain pathology between PKU and neurodegenerative diseases may be considered cause for extra attention to the aging process in PKU.

Whereas currently myelin damage and monoaminergic neurotransmitter and precursor deficiencies appear the most likely biomarkers for PKU pathology as they have both been related, though mostly separately, to elevated Phe levels and to specific cognitive impairment, it is important, particularly for the monoamine-based hypotheses, to consider neurobiological connectivity, or the dynamic interplay between biomarker systems [108,121,122,123]. Whereas myelin damage has almost automatically been related to structural and functional connectivity between brain regions, this is not so clearly the case for neurobiological connectivity, possibly because of its high complexity. Despite the rise of metabolomics and lipidomics studies to identify relevant biomarkers, most (human) PKU studies to date have focused on a pre-established selection of biomarkers: phenylalanine, obviously, dopamine precursor tyrosine, and serotonin precursor tryptophan. While this is changing and more candidate biomarkers (enzymes, cofactors, metabolites) are added to the PKU metabolic profile, it is pivotal to look beyond the different pathways involved directly in Phe metabolism. For PKU, there are tyrosine-enriched food supplements in addition to the Phe-restricted diet, but it is not fully clear to what extent this affects the intake of other nutrients [124]. Moreover, disrupted metabolic activity in PKU costs energy (i.e., glucose) to mitigate disruptions, which can thus possibly be used less for other aspects of brain functioning underlying several cognitive outcomes (especially sustained attention) [125,126].

Regardless of the abovementioned important considerations, the commonalities in affected metabolic pathways and neurotransmitter abnormalities, as well as cognitive-behavioral phenotypes between PKU and different neurodevelopmental disorders and neurodegenerative diseases, provide a further basis for investigating and monitoring exactly those phenotypes across the lifespan in PKU.

## 8. Nutrients and Cognition in PKU, Neurodevelopmental Disorders, and Neurodegenerative Diseases

PKU is increasingly treated with pharmaceuticals, but the mainstay of treatment has been, and still is, a low-Phe diet, supplemented with LNAAs (particularly tyrosine). Also, different pharmaceutical treatments are quite successful in lowering Phe levels, which, in turn, often leads to dietary liberalization [21,22,23]. There are many nutrients that have been related to brain functioning and cognitive outcomes, and even more that have convincingly been related to physical/physiological health outcomes (which could subsequently affect wellbeing and cognition). And it seems that both the adherence to dietary or pharmacological treatment or lack thereof in PKU could cause shortages or fluctuations in several nutrient levels or systems influenced by fluctuating nutrient levels, such as the microbiota-gut-brain axis, e.g., [127]. For example, Cannet and colleagues used NMR spectroscopy and quantified 105 lipoprotein parameters (including lipoprotein subclasses) and 24 low molecular weight parameters to assess whether there were Phe-associated abnormalities in (Low Density Lipoprotein; LDL-) cholesterol synthesis and regulation in adult PKU patients (mean age 38.7 years) [128]. They were specifically interested in (LDL-)cholesterol because of its supposed roles in the development of atherosclerosis and in membrane structure and myelination. Total cholesterol and LDL-cholesterol (as well as 22 LDL subclasses) were significantly lower in PKU patients than in controls (and these levels were negatively correlated with concurrent Phe). Tyrosine, glutamine, and creatinine were also significantly lower in PKU patients compared to controls, while citric and glutamic acids were significantly higher. The lipoprotein profile of PKU could, therefore, be protective against atherosclerosis, but it might have negative effects, particularly in combination with the non-lipoprotein changes in the metabolic profile, on membrane structures and myelination, which, in turn, might affect cognition.

Dietary management of PKU not only focuses on restricting Phe intake and supplementation of tyrosine, but it also aims to achieve adequate nutrition in full [129,130]. In a comprehensive review, Montoya-Parra and colleagues described nutrients known to fulfill a role in brain development and functioning (nutrients: Long-Chain Polyunsaturated Fatty Acids (LC-PUFAs) such as docosahexaenoic acid (DHA) and ecosapentaenoic acid (EPA), selenium, vitamins B6, B12, E, C, A, D, folic acid, choline, uridine, calcium, magnesium, zinc, iron, iodine and cholesterol). Comparisons between PKU patients and healthy controls showed significantly lower levels of DHA, EPA, and cholesterol for PKU patients [130].

The authors further concluded that there may be more nutrients that are deficient in PKU through insufficient dietary intake, but called for more research before making such claims. For the LC-PUFAs (DHA, EPA), supplementation studies provided further evidence for a (beneficial) role in cognitive functioning of PKU patients, as did LNAA-supplementation studies [131,132,133]. For example, Scala and colleagues treated 10 adult PKU patients with poor metabolic control for 12 months with LNAAs and found no worsening in plasma Phe, an improvement in Tyr levels, and concurrent improvements of distress and well-being rates and of cognition (executive functions and sustained attention) [133]. Supplementation with Glycomacropeptide (GMP), a low-Phe protein derived from cheese whey serving as a more palatable alternative to traditional amino acid-based medical foods, and micronutrients such as Folate and Vitamin B12, have, to date, shown some promising effects, although these do not yet, or only speculatively or indirectly, extend to cognitive functioning in PKU [134].

In correspondence with our earlier plea to attend to older PKU patients because of the risk of accelerated aging and cognitive decline, similarities can be observed between nutrients and LNAAs that were shown to be reduced in PKU and those that may be depleted and benefit from supplementation in neurodegenerative diseases. Particularly, supplementation, or at least a high intake of LC-PUFAs (DHA, EPA), has been associated with a reduced risk of cognitive decline and dementia [135,136]. Although without strong evidence for PKU as of yet, elevated homocysteine levels, linked to deficiencies in magnesium, folate, and vitamin B12, have been associated with an increased risk of dementia and cognitive decline. Supplementation with these vitamins may help lower homocysteine levels and support cognitive health. LNAA tyrosine is the metabolic precursor of dopamine, and could be considered for supplementation in PD (where dopamine is significantly depleted). And vitamins C and E, although not yet clearly involved in either PKU or neurodegenerative diseases, are antioxidants and may protect against oxidative stress, a supposedly contributing factor to cognitive problems in both PKU and neurodegenerative diseases [137,138,139].

Also, modest benefits of free fatty acid supplementation were observed in neurodevelopmental disorders such as ADHD, reducing its symptoms (although artificial food color exclusion seemed to produce even larger effects) [140]. As is generally recognized, it is a realistic possibility that many of these supplements benefit most, if not all, individuals. Still, unless specific neurobiological characteristics of particular disorders interfere with their effectiveness, they might help improving cognition and well-being of individuals with particular disorders, such as PKU, over and above targeted supplementation (such as tyrosine and tryptophan in PKU).

Thus, through shared mechanisms such as precursor availability, neurotransmitter balance, and nutritional support, PKU, neurodevelopmental disorders (such as ADHD), and neurodegenerative diseases (such as AD and PD) converge in neurochemical pathways (and brain integrity) underpinning executive and attentional cognition. Such shared biomarkers also provide a much larger knowledge platform or basis for treatments and interventions across disorders and diseases [127,141,142].

## 9. Conclusions

In summary, there is a clear need for more comprehensive research focusing on the cognitive functioning of very young and aging PKU patients. As our understanding of the life-long implications of PKU evolves, it is essential to consider the potential for cognitive decline and the risk factors that may emerge in aging populations. Further studies should aim to elucidate the relationships between PKU and neurodegenerative conditions regarding executive functioning, neurotransmitter dysregulation, and structural brain abnormalities, as well as the influence of nutrients on those processes. Together with a more thorough investigation of cognition during early life stages and its influence on later development, this will ultimately inform more effective intervention strategies for individuals with PKU across the lifespan.

It is also concluded that there are still quite a few challenges that demand attention in future studies investigating very young and older or aging PKU patients. The most consistently vulnerable areas of cognition in PKU patients are executive functioning and sustained attention, with several factors influencing the likelihood of those showing group differences or associations with metabolic control. These factors include whether the core executive functions (inhibitory control, working memory and cognitive flexibility) are tested in isolation or in combination, whether they are tested in combination with other cognitive functions (such as sustained attention or motor control), whether speed or accuracy (or both) are tested, whether they are tested at a basic or more complex level, and possibly, whether testing occurs in an emotional or motivational context. A number of apparently suitable tasks and test batteries were listed in this review. However, there are also factors such as the age of the participants that are likely to influence whether group differences or associations with metabolic control are found. This review provided neuropsychological testing options and considerations for PKU patients of all ages.

Not only cognition and how it is tested, particularly in very young and aging PKU patients, deserve increased attention in future studies. Another important topic to consider is the monitoring of metabolic control and nutritional status over time. This extends beyond monitoring selected amino acids (e.g., phenylalanine, tyrosine, tryptophan). Future studies should strive toward conjunctive monitoring of amino acids, other nutrients and proteins, and neurotransmitters in relation to cognition and mental health.

## Figures and Tables

**Table 1:** Executive Function Assessment Across Age Groups.

Age Group	Executive Function Tasks	Notes
Infancy (0–2 years)	IC^†^ Early inhibitory/self-regulatory tasks (“Don’t” paradigm)	To be supplemented by Bayley Scales of Infant and Toddler Development
Early Childhood (3–5 years)	IC^†^ Day–Night Stroop; Luria’s Hand Game; “Simon Says” WM^‡^ Spatial/Digit Span; Hide-and-Seek WM tasks CF^§^ Dimensional Change Card Sort (DCCS) Preschool ^3^; Brixton Preschool	To be supplemented by early language tasks and early motor tasks Age range where emotional/motivational components can be introduced and hot and cool EF can be contrasted
Middle Childhood (6–12 years)	IC^†^ Go/No-Go ^1^; Flanker ^3^ WM^‡^ N-back ^1^; RVP/RVIP ^1^; Spatial/Digit Span ^1^; Memory Search 2D ^2^; List Sorting WM ^3^ CF^§^ Multitasking Test (MTT) ^1^; Shifting Set–Visual ^2^; DCCS ^3^	Age range where mixed EF tasks (e.g., IC + sustained attention ^2^, CF + motor ^2^) can be introduced
Adolescence (13–18 years)	IC^†^ Go/No-Go ^1^; Flanker ^3^ WM^‡^ N-back ^1^; RVP/RVIP ^1^; Spatial/Digit Span ^1^; Memory Search 2D ^2^; List Sorting WM ^3^ CF^§^ MTT ^1^; Shifting Set–Visual ^2^; DCCS ^3^	
Adulthood (18–50 years)	IC^†^ Go/No-Go ^1^; Flanker ^3^ WM^‡^ N-back ^1^; RVP/RVIP ^1^; Spatial/Digit Span ^1^; Memory Search 2D ^2^; List Sorting WM ^3^ CF^§^ MTT ^1^; Shifting Set–Visual ^2^; DCCS ^3^	
Older Adults (50+ years)	IC^†^ Go/No-Go ^1^; Flanker ^3^ WM^‡^ N-back ^1^; RVP/RVIP ^1^; Spatial/Digit Span ^1^; Memory Search 2D ^2^; List Sorting WM ^3^ CF^§^ MTT ^1^; Shifting Set–Visual ^2^; DCCS ^3^	To be supplemented by episodic and semantic memory tasks and motor tasks ^4,5,6^ Screening instruments contain further non-digitized EF tasks, such as ‘Three-step command’ ^6^ (IC^†^), ‘Serial 7s’ ^6^ (WM^‡^), ‘Trail Making Test’ ^5^ (CF^§^), and ‘Verbal Fluency’ ^5^ (CF^§^)

Executive Function Codes: IC^†^ = Inhibitory Control. WM^‡^ = Working Memory. CF^§^ = Cognitive Flexibility. Computerized Battery Codes: ^1^ CANTAB—Cambridge Neuropsychological Test Automated Battery. ^2^ ANT—Amsterdam Neuropsychological Tasks. ^3^ NIH Toolbox—National Institutes of Health Toolbox Cognitive Battery. Screening Instruments: ^4^ MoCA—Montreal Cognitive Assessment, ^5^ ACE-III—Addenbrooke’s Cognitive Examination–III, ^6^ MMSE—Mini-Mental State Examination. Table 1 contains suggestions for EF-assessment across ages. It is not meant to be exhaustive or conclusive regarding neuropsychological instruments that can be used towards this aim. Age groups are informed choices: exact cut-off ages for different groups cannot be claimed.

## Data Availability

No new data were created or analyzed in this study. Data sharing does not apply to this article.
